# The development and psychometric evaluation of specific problem lists reflecting psychosocial distress of patients before and after solid organ transplantation

**DOI:** 10.3389/fpsyg.2025.1481641

**Published:** 2025-05-13

**Authors:** Evamaria Müller, Martin Härter, Sanna Higgen, Markus J. Barten, Doreen Eickhoff, Florian Grahammer, Nele Reinsberg, Martina R. Sterneck, Angela Buchholz

**Affiliations:** ^1^Department of Medical Psychology, University Medical Center Hamburg-Eppendorf, Hamburg, Germany; ^2^Department of Cardiovascular Surgery, University Medical Center Hamburg-Eppendorf, Hamburg, Germany; ^3^Center for Internal Medicine, University Medical Center Hamburg-Eppendorf, Hamburg, Germany; ^4^Department of Human Medicine, Faculty of Medicine, MSH Medical School Hamburg, Hamburg, Germany; ^5^MSH Medical School Hamburg, ICAN Institute for Cognitive and Affective Neuroscience, Hamburg, Germany; ^6^Department of Internal Medicine, University Medical Center Hamburg-Eppendorf, Hamburg, Germany; ^7^University Transplant Center, University Medical Center Hamburg-Eppendorf, Hamburg, Germany

**Keywords:** end-stage disease, solid organ transplantation, psychosocial distress, transplant recipients, screening

## Abstract

**Introduction:**

Psychosocial distress is common in patients before and after solid organ transplantation. Regular screening facilitates the early identification of distressed patients and the provision of appropriate professional care. However, feasible screening tools that address the specific problems of transplant patients are missing. Thus, the aim of this mixed methods study was to develop transplant-specific problem lists for patients before (transplant candidates) and after (transplant recipients) solid organ transplantation which can be used as a quick and easily applicable screening tool for psychosocial distress.

**Method:**

An electronic database search resulted in a preliminary item list including 36 problems common in transplant candidates and 44 problems in transplant recipients. A total of *N* = 117 patients and *N* = 48 health care providers participated in a paper-pencil survey to assess the relevance and comprehensibility of the problem lists. Qualitative interviews about the clarity and completeness of problem lists were performed with *N* = 58 patients and *N* = 3 transplant nurses. Data analysis included the calculation of descriptive statistics and content analysis of interviews and survey open response fields. To test the concurrent validity of the problem list for patients, patients completed the problem list in combination with the National Comprehensive Cancer Network (NCNN) distress thermometer and a short form of the Patient Health Questionnaire (PHQ-4) as part of routine care.

**Results:**

The finalized list for transplant candidates includes 21 items and the list for transplant recipients 22 items, each covering four categories: problems in everyday life, social problems, worries and anxieties, physical and psychological problems. In the course of the study, sufficient data was gathered only from transplant recipients (*N* = 100). The number of problems endorsed by transplant recipients correlated significantly with measures of depression and distress (distress: *r* = 0.41, *p* < 0.001; PHQ-4: *r* = 0.63, *p* < 0.001; PHQ-2: *r* = 0.53, *p* < 0.001; GAD-2: *r* = 0.60, *p* < 0.001). The developed problem lists cover relevant psychosocial problems and can help to identify distress in patients before and after transplantation.

**Discussion:**

The problem list for transplant recipients showed sufficient concurrent validity, psychometric properties of the problem list for transplant candidates should be investigated in further studies.

## Introduction

In 2023, 3,646 solid organ transplantations were performed in Germany and 8,387 patients were waiting to receive an organ (Deutsche Stiftung Organtransplantation, 2023). Compared to the general population, the prevalence of mental disorders is substantially higher in patients throughout the whole process of solid organ transplantation (SOT) from evaluation of eligibility for SOT to long-term survival after transplantation (Evans et al., 2015; Rosenberger et al., 2012). As one example, depression is one of the most common mental disorders in transplant patients (Heinrich and Marcangelo, 2009; Baumann et al., 2019) and has a negative impact on several transplant outcomes (Kugler et al., 2013). Numerous studies showed that post-Tx depression was strongly associated with higher morbidity and mortality in transplant patients (Heinrich and Marcangelo, 2009; Rosenberger et al., 2012; Corbett et al., 2013; Dew et al., 2015; de Zwaan et al., 2023; Smith et al., 2016; Rogal et al., 2013). This is also emphasized by the new German clinical practice guideline on the psychosocial diagnosis and treatment of patients before and after organ transplantation. The authors underline the need for a multidisciplinary approach including screening, diagnostics and treatment by mental health professionals (de Zwaan et al., 2023).

Besides a high prevalence of mental disorders, transplant patients also suffer from a range of problems referring to their daily life, medical symptoms and management of their disease such as physical and psychological trauma, hopelessness, fear of the future, fear of death, questions around the meaning of life (Novak et al., 2013). The most appropriate concept to capture such problems beyond the criteria of a mental disorder is the concept of psychosocial distress. Psychosocial distress summarizes a range of extra-ordinary, troubling or confusing symptoms and/or experiences that lead to emotional suffering (Drapeau et al., 2012; Baranyi et al., 2013). The National Comprehensive Cancer Network (NCNN) defines distress as a “multifactorial unpleasant experience of a psychological (i.e., cognitive, behavioral, emotional), social, spiritual, and/or physical nature that may interfere with the ability to cope effectively with cancer, its physical symptoms, and its treatment” (Riba et al., 2019). This definition of psychological distress for cancer patients seems equally appropriate for patients facing other life-threatening chronic illnesses involving lifelong medical treatment with a serious impact on daily life such as transplant patients.

Psychosocial distress is common in transplant patients (Heinrich and Marcangelo, 2009; Kuntz et al., 2015) and impairs their quality of life (Baranyi et al., 2013; Novak et al., 2013), including anxiety, stress, worry, panic and fear (Chad-Friedman et al., 2017). If left untreated, it may contribute to the development of mental disorders like depression (Drapeau et al., 2012). Early identification and treatment of transplant patients suffering from psychosocial distress may improve on the long hand meaningful outcomes such as quality of life (Baranyi et al., 2013; Kugler et al., 2013; Miller et al., 2013), adherence to medication (Achille et al., 2006), as well as morbidity and mortality (Novak et al., 2010; DiMartini et al., 2011; Smith et al., 2016). Having this in mind, regular screening for psychosocial distress throughout the whole transplantation process is highly recommended (Heinrich and Marcangelo, 2009; DiMartini et al., 2011; Rosenberger et al., 2012; Kugler et al., 2013; Miller et al., 2013; Novak et al., 2013; Rogal et al., 2013; Dew et al., 2015).

As a significant proportion of physicians do not address emotional problems in patient-physician consultations (Mitchell et al., 2008) and tends to underestimate psychosocial distress (Dew and DiMartini, 2005), screening tools are promising means to identify patients that require professional help (Carlson and Bultz, 2003; Rosenberger et al., 2016). To facilitate regular screening, brief and easy-to-use screening tools are needed (Dew and DiMartini, 2005; Novak et al., 2013) that address the specific concerns of transplant patients and provide health care providers (HCPs) with important information (Grady et al., 2009; Li et al., 2012).

Until today, there is no consensus on how to measure psychosocial distress. Oftentimes it is assessed using instruments designed to assess symptoms of depression or anxiety (Alanazi et al., 2023). However, this approach does not reflect the broad definition developed by the NCCN deliberately going beyond the recording of symptoms of a mental disease. Based on their broad concept of psychosocial distress, the NCNN distress thermometer has been proposed. It is an oncology-specific screening instrument that is used worldwide to identify cancer patients with increased levels of psychosocial distress (Donovan et al., 2014) and includes a cancer-specific problem list. It is essential that the problem list summarizes the problems most relevant to patients (Brennan et al., 2012). For transplant patients, a comparable tool is missing.

The present study aimed to develop problem lists reflecting those problems that are considered most relevant for transplant patients in different stages of the transplantation process. Since the transplant patients’ perspective was considered as being most important at this stage of development, our main focus was content-validity (Terwee et al., 2018). The second aim was to gather preliminary data regarding the concurrent validity. Since transplant candidates face different psychosocial challenges compared to transplant recipients (Köllner and Archonti, 2003) it was decided at the beginning of the project to develop two distinct problem lists for these two stages of the transplant process. Both problem lists refer to the situation of transplant patients, living donors are not addressed in our study.

## Materials and methods

### Study design and setting

We conducted a mixed methods study including an electronic database search, paper-pencil surveys and individual interviews with transplant patients and health care providers (HCPs) working with transplant patients at the University Medical Center Hamburg-Eppendorf (UKE). Additionally, an observational design was applied. The study was carried out at the Hamburg-Eppendorf University Transplant Center (UTC), which is one of the largest German transplant centers. The UTC consists of several inpatient and outpatient facilities treating transplant patients at all stages of the transplantation process (liver, lung, kidney, heart) and is located at the UKE.

### Ethical approval

The study was carried out in accordance with the Code of Ethics of the Declaration of Helsinki and was approved by the Local Ethics Committee of the Center for Psychosocial Medicine, UKE, Germany (Registration Code LPEK-0029).

### Participants

Transplant patients were invited to take part in different parts of our study during regular visits of different units of the UTC. This is, reasons why the patients entered the UTC could be very different varying from short control visits to longer inpatient treatment episodes. Aim of this procedure was to increase variability of perspectives at this early stage of development. Adult patients who were listed for (candidates) or had already received SOT (recipients; liver, lung, heart, kidney), spoke German sufficiently and were sufficiently healthy, were eligible for study participation. Patients were not included when they were to weak to participate or had insufficient language skills.

### Development of preliminary psychosocial problem lists

Content validity consist of the three aspects relevance, comprehensiveness and comprehensibility (Terwee et al., 2018). In order to identify all relevant psychosocial problems of patients before and after solid organ transplantation (heart, lung, liver, kidney) an electronic database search in PubMed was performed. The following search terms were used: organ transplantation/psychology and (psychosocial* or psychological*) (all fields); Transplantation/psychology and kidney and distress (all fields); transplantation/psychology and liver and, psychosocial*“(all fields); organ transplantation/psychology and (psychosocial* or psychological* or psychiatric*) and (heart* or lung*). Additionally, the “similar articles” function in PubMed was used to identify articles that fit the purpose. Thirty-six articles were screened for relevant psychosocial problems and discussed in the research team. Two team members (EM and NR) clustered psychosocial problems relevant for transplant candidates and recipients. This resulted in a preliminary problem list for transplant candidates including 36 problems clustered in six categories and a preliminary problem list for transplant recipients including 44 problems clustered in seven categories (see Supplementary File S1).

### Data collection

According to the COnsensus-based Standards for the selection of health Measurement INstruments (COSMIN) patients and professionals should be asked about the relevance, comprehensiveness and comprehensibility of the PROM items to assess the content validity (Terwee et al., 2018). Data collection took place over a period of 7 weeks from May 2019 until July 2019 in six hospital wards and two outpatient clinics of the University Transplant Center (UTC) located at the UKE and included patients and HCPs. Members of the study team (EM and NR) approached hospitalized patients and patients visiting clinics and invited them to participate. After signing an informed consent sheet, patients were asked to reply to questionnaires assessing the comprehensibility and relevance of the problem lists for candidates or recipients depending on their current Tx status. Patients were asked to mark items they considered unclear and to rate how relevant they found the problems on a 4-point scale (1 = not at all relevant, 4 = very relevant) based on their own experience. The questionnaires included open response fields for missing problems and general comments. Patients were also asked to provide sociodemographic data, information about their medical Tx history and to rate their general and mental health status on a scale ranging from 1 (poor) to 5 (excellent). Afterwards, patients were asked to participate in a short audio-recorded interview, if they found the wording of some problems unclear, mentioned missing problems, or showed an interest in discussing the problems from their point of view.

Likewise, HCPs, including medical assistants, nurses, psychologists, assistant physicians, senior physicians working in the University Transplant Center (UTC) at the UKE were also invited to participate in the paper-pencil survey. They were also asked to sign an informed consent sheet and fill out the questionnaires assessing the comprehensibility and relevance of the items for transplant candidates and transplant recipients based on their own experience with transplant patients and to provide sociodemographic data and information about their medical expertise. Four transplant nurses of the UTC were asked to participate in individual interviews about the preliminary psychosocial problem lists.

### Psychometric assessment

This second study part was conducted at the outpatient clinic for heart failure, heart and lung transplantation and artificial heart systems and on the transplant ward for visceral transplant surgery of the UTC. Patients who were currently being treated in one of the participating units were eligible to participate. This data was assessed as part of the pilot implementation of the problem lists. The study description and results of the implementation study are published elsewhere (Higgen et al., 2025).

Patients reported some sociodemographic data (age, gender, highest grade of education, current professional status). Patients replied to a single question on their general health and their mental health on a 5-point Likert-scale (1 “bad” to 5 “excellent”). The problem list was embedded into a screening consisting of four parts:

1)   The NCCN distress thermometer (25) to measure psychosocial distress (from 0 to 10).
2)   The newly developed problem list for SOT recipients.
3)   The short form of the Patient Health Questionnaire (PHQ-4) to measure depressive and anxiety symptoms (36). An overall sum-score for the PHQ-4 as well as sum-scores for the subscales PHQ-2 (depression) and GAD-2 (anxiety) can be calculated. The PHQ-4 is scored as follows: normal (0–2), mild (3–5), moderate (6–8) and severe (9–12).
4)   A question to indicate whether the patient would like to talk to a psychologist.

Patients that were willing to participate obtained a consent form and the screening. Inpatients received the screening from a transplant psychologist. Outpatients received the screening from an HCP during their regular check-ups.

### Selection and adaptation of psychosocial problems

First, descriptive statistics of the quantitative survey data were used to identify psychosocial problems relevant to pre-Tx and post-Tx patients. Based on a weighting of economy and completeness the following criteria for inclusion were determined: (1) a minimum of 15 HCPs rated the problem as “very relevant,” (2) the mean of HCP relevance ratings reached 3 or more, (3) a minimum of 10% of pre-or post-Tx patients, respectively, rated the problem as “very relevant,” (4) the mean of patient relevance ratings reached 2.5 or more, (5) the mean of young (<40 years old) patient relevance ratings reached 2.5 or more. Problems were considered for inclusion in the problem lists, if at least two of the criteria were met. Second, results from the qualitative analysis of the interviews and open response fields of the survey questionnaires were used to adapt problems that were rated as unclear in the survey, and to identify missing problems. Three researchers (EM, NR, and AB) discussed the adaptation and wording of the problem lists and agreed on the final versions.

### Data analysis

Descriptive statistics were calculated to analyze survey data using the Statistical Package for the Social Sciences (SPSS version 23, IBM, Armonk, United States). Audio-recorded interviews were transcribed using the program the transcription software F4 (version F4.2, dr. dresing & pehl GmbH, Marburg, Germany). Analysis of interview transcripts was done with MAXQDA (version MAXQDA 10, VERBI GmbH, Berlin, Germany) and Excel software (Excel 2013, Microsoft Corporation, Redmond, United States). Two researchers (EM and NR) performed qualitative analysis. Referring to the aims of the interviews to discuss clarity and completeness of the psychosocial problem lists, a broad category system was derived deductively. Thirty percent of the transcripts were coded independently by EM and NR using MAXQDA software. The codings were compared and discussed until agreement was reached. The same procedure was applied to the remaining 70% of the transcripts. Broad category codings were transferred to Excel software and subcategories were developed inductively based on the content of the interview transcripts. Subcategory development and coding was done by EM and NR together.

Correlations between the number of problems endorsed by patients and the NCNN distress thermometer, the PHQ-4, PHQ-2, and GAD-2, the general health, the mental health and the need to talk were calculated to evaluate the concurrent validity of the problem list. The level of distress was dichotomized using a cut of value of ≥4 (Jacobsen et al., 2005; Donovan et al., 2014). A significance level of *p* < 0.05 was set.

## Results

### Sample characteristics (development phase)

**Figure 1:**
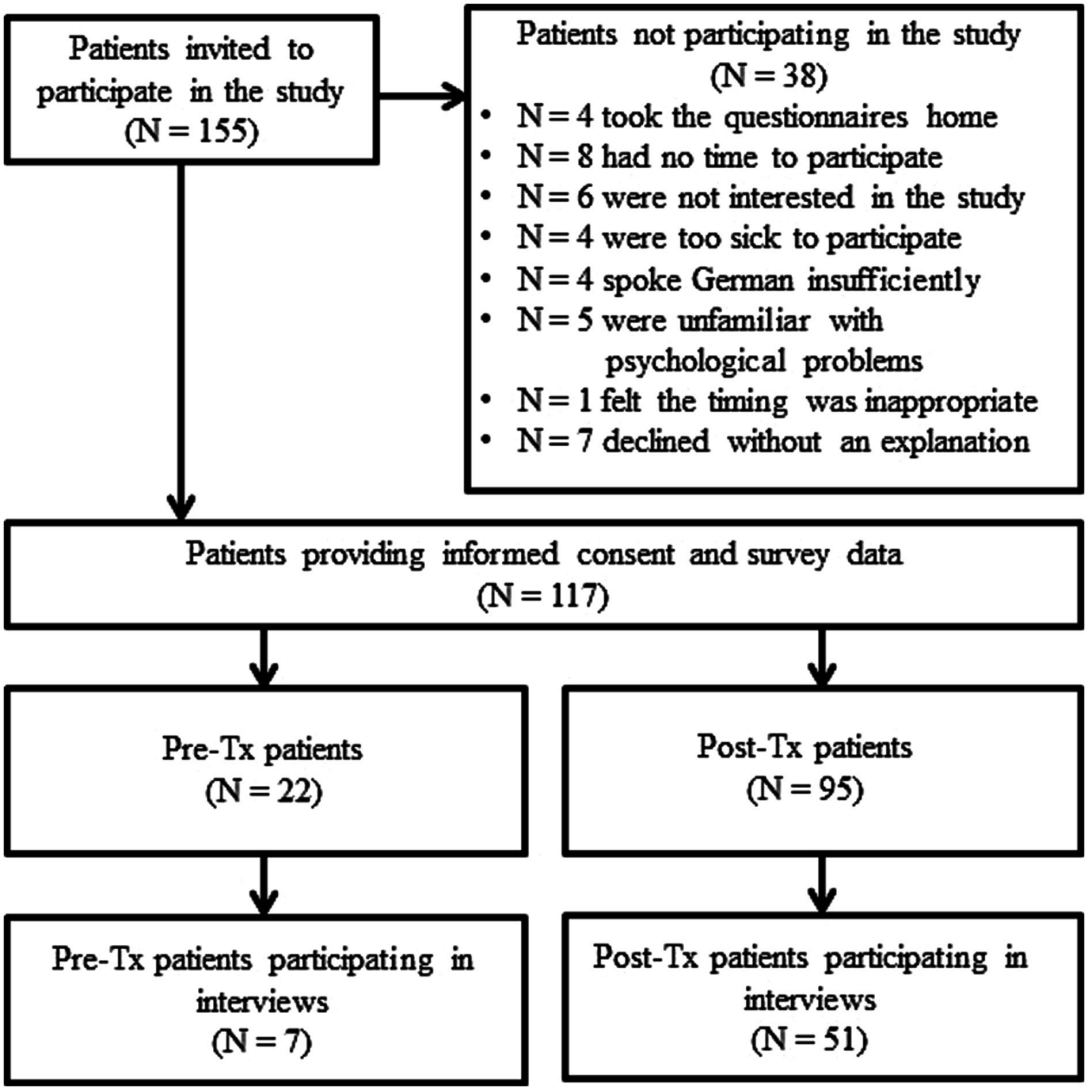
Patient flow of participation.

Of the *N* = 155 patients invited to participate in the survey, *N* = 117 gave informed consent and provided survey data for analysis. Altogether *N* = 38 declined participation in the study for various reasons (see Figure 1 Patient flow of participation). Of the *N* = 117 patients participating in the survey, *N* = 22 were pre-Tx patients and *N* = 95 were post-Tx patients. Participating patients were predominantly male (*N* = 72, 62%) outpatients (*N* = 80, 68.4%) and had a mean age of 55.25 years (SD = 12.47, range: 24–84 years). Patient ratings of their general health status had a mean of 3.01 (SD = 0.80), ratings of their mental health status had a mean of 3.28 (SD = 0.93). Further details on pre-Tx and post-Tx patients are represented in Tables 1, 2, respectively. Of the *N* = 140 HCPs invited to participate in the survey, *N* = 48 provided informed consent and replied to the questionnaires. Reasons for non-participation are unknown. Most participating HCPs were female (75%), worked in the care of liver or kidney transplant patients (64.6%) and had 5 or more years of experience working with Tx patients (52%). The mean age of participating HCPs was 38.1 years (SD = 9.20, range: 22–56 years). Further details are depicted in Table 3. Of the *N* = 4 transplant nurses invited to participate in interviews, *N* = 3 participated. They were female, had a mean age of 50 years (SD = 4.36, range: 45–53 years) and more than 5 years of experience working with transplant patients.

**Table 1:** Sociodemographic data of transplant candidates.

Transplant candidates	Total	Location		Organ			
		Inpatients	Outpatients	Liver	Kidney	Heart	Lung
Total *N*	22	14	8	8	2	11	1
Age, mean (SD)	51.77 (10.84)	51.21 (12.32)	52.75 (8.28)	48.75 (12.44)	49.50 (23.34)	53.09 (7.30)	66 (n/a)
Gender, *N* (%)							
Female	11 (50)	7 (50)	4 (50)	5 (62.5)	0 (0)	5 (45.5)	1 (100)
Male	11 (50)	7 (50)	4 (50)	3 (37.5)	2 (100)	6 (54.5)	0 (0)
Highest grade of education, *N* (%)							
No school-leaving certificate	1 (4.5)	1 (7.1)	—	1 (12.5)	—	—	—
Certificate of secondary education	5 (22.7)	5 (35.7)	—	—	2 (100)	3 (27.3)	—
General certificate of secondary education	4 (18.2)	3 (21.4)	1 (12.5)	2 (25)	—	2 (18.2)	—
University of applied sciences entrance qualification	2 (9.1)	1 (7.1)	1 (12.5)	1 (12.5)	—	1 (9.1)	—
General qualification of university entrance	7 (31.8)	3 (21.4)	4 (50)	2 (25)	—	4 (36.4)	1 (100)
University degree	3 (13.6)	1 (7.1)	2 (25)	2 (25)	—	1 (9.1)	—
Other	—	—	—	—	—	—	—
Current professional status, *N* (%)							
Working	9 (40.9)	5 (35.7)	4 (50)	5 (62.5)	1 (50)	3 (27.3)	—
Retired	9 (40.9)	6 (42.9)	3 (37.5)	1 (12.5)	1 (50)	6 (54.5)	1 (100)
Home keeper	—	—	—	—	—	—	—
Pupil/student/in training	—	—	—	—	—	—	—
Unemployed	3 (13.6)	2 (14.3)	1 (12.5)	1 (12.5)	—	2 (18.2)	—
Other	1 (4.5)	1 (7.1)	—	1 (12.5)	—	—	—
Year of listing ≤2017, *N* (%)	7 (33)	3 (23)	4 (50)	3 (62.5)	1 (100)	2 (18)	1 (100)
Year of listing ≥2018, *N* (%)	14 (67)	10 (77)	4 (50)	5 (37.5)	—	9 (82)	—
General health, mean (SD)	2.59 (0.67)	2.5 (0.65)	2.75 (0.71)	3 (0.54)	3 (0)	2.27 (0.65)	2 (n/a)
Mental health, mean (SD)	3.18 (0.8)	3.14 (0.78)	3.25 (0.89)	3.38 (1.06)	3.5 (0.71)	3 (0.63)	3 (n/a)

SD, standard deviation; Tx, transplant.

**Table 2:** Sociodemographic data of transplant recipients including patients after implementation of the screening.

Transplant recipients	Total	Location		Organ				Total after implementation
		Inpatients	Outpatients	Liver	Kidney	Heart	Lung	
Total *N*	95	23	72	36	32	18	9	100
Age, mean (SD)	56.06 (12.73)	54.96 (15.07)	56.42 (11.98)	57.44 (13.36)	54.63 (12.21)	55.61 (13.84)	56.62 (10.73)	98 (98)
Gender, *N* (%)								
Female	34 (35.8)	7 (30.4)	27 (37.5)	11 (30.6)	14 (43.8)	3 (16.7)	6 (66.7)	43 (43)
Male	61 (64.3)	16 (69.6)	45 (62.5)	25 (69.4)	18 (56.3)	15 (83.3)	3 (33.3)	57 (57)
Highest grade of education, *N* (%)								100 (100)
No school-leaving certificate	3 (3.2)	1 (4.3)	2 (2.8)	1 (2.8)	1 (3.1)	—	1 (11.1)	9 (9)
Certificate of secondary education	24 (25.3)	7 (30.4)	17 (23.6)	9 (25)	9 (28.1)	4 (22.2)	2 (22.2)	—
General certificate of secondary education	30 (31.6)	7 (30.4)	23 (31.9)	11 (30.6)	9 (28.1)	8 (44.4)	2 (22.2)	52 (52)
University of applied sciences entrance qualification	5 (5.3)	—	5 (6.9)	4 (11.1)	—	1 (5.6)	—	—
General qualification of university entrance	9 (9.5)	1 (4.3)	8 (11.1)	3 (8.3)	4 (12.5)	2 (11.1)	—	20 (20)
University degree	20 (21.1)	7 (30.4)	13 (18.1)	6 (16.7)	8 (25)	3 (16.7)	3 (33.3)	18 (18)
Other	4 (4.2)	—	4 (5.6)	2 (5.6)	1 (3.1)	—	1 (11.1)	1 (1)
Current professional status, *N* (%)								99 (99)
Working	27 (28.4)	7 (30.4)	20 (27.8)	9 (25)	9 (28.1)	8 (44.4)	1 (11.1)	32 (32.3)
Retired	58 (61.1)	12 (52.2)	46 (63.9)	24 (66.7)	18 (56.3)	9 (50)	7 (77.8)	53 (53.5)
Home keeper	1 (1.1)	—	1 (1.4)	—	1 (3.1)	—	—	1 (1)
Pupil/student/in training	—	—	—	—	—	—	—	1 (1)
Unemployed	4 (4.2)	3 (13)	1 (1.4)	—	3 (9.4)	—	1 (11.1)	8 (8.1)
Other	5 (5.3)	1 (4.3)	4 (5.6)	3 (8.3)	1 (3.1)	1 (5.6)	—	4 (4)
Year of transplantation ≤2017, *N* (%)	61 (64.2)	9 (39)	52 (72)	24 (67)	17 (53)	14 (78)	6 (66.7)	—
Year of transplantation ≥2018, *N* (%)	34 (35.8)	14 (61)	20 (28)	12 (33)	15 (47)	4 (22)	3 (33.3)	—
General health, mean (SD)	3.11 (0.81)	2.96 (0.71)	3.15 (0.83)	3.22 (0.96)	2.81 (0.47)	3.11 (8.32)	3.67 (0.71)	3.02 (0.90)
Mental health, mean (SD)	3.30 (0.96)	3.52 (0.73)	3.23 (1.02)	3.42 (0.94)	3 (0.86)	3.28 (1.07)	3.89 (0.93)	3.18 (1.01)

SD, standard deviation; Tx, transplant. The sample after implementation are the patients included in the psychometric assessment.

**Table 3:** Sociodemographic data of health care providers.

Total *N*	48
Age, mean (SD)	38.07 (9.20)
Gender, *N* (%)	
Female	36 (75)
Male	12 (25)
Professional background, *N* (%)	
Nurse	17 (35.4)
Medical assistant	3 (6.3)
Psychologist	3 (6.3)
Junior physician	11 (22.9)
Senior physician	10 (20.8)
Other	4 (8.3)
Current Tx workplace, *N* (%)	
Heart or lung Tx patients	17 (35.4)
Liver or kidney Tx patients	31 (64.6)
Inpatient ward	16 (33.3)
Outpatient clinic	32 (66.7)
Current contact with Tx patients, *N* (%)	
Yes	43 (89.6)
No	5 (10.4)
Professional experience with Tx patients, *N* (%)	
<5 years	23 (47.9)
5–10 years	10 (20.8)
11–20 years	11 (22.9)
>20 years	4 (8.3)

SD, standard deviation; Tx, transplant.

### Selection of psychosocial items for the problem list

Mean relevance ratings for psychosocial problems before solid organ transplantation by pre-Tx patients ranged between 1.32 (SD = 0.72) for *decisional conflict about the transplantation* and 2.82 (SD = 0.96) for *concern about the donor organ arriving in time*. Mean relevance ratings by HCPs ranged between 2.51 (SD = 0.84) for *problems with self-esteem* and 3.74 (SD = 0.49) for *strain due to the uncertain waiting period*. Detailed results are presented in Table 4. Mean relevance ratings for psychosocial problems after solid organ transplantation by post-Tx patients ranged between 1.39 (SD = 0.84) for *conflicts with health care providers* and 3.38 (SD = 0.96) for *feelings of responsibility for the new organ*. Mean relevance ratings by HCPs ranged between 1.93 (SD = 0.93) for *mourning over the lost organ* and 3.66 (SD = 0.57) for *fear of organ rejection*. Detailed results are presented in Table 5. The application of the selection criteria described in the methods section above reduced the number of psychosocial problems to 20 problems for pre-Tx and post-Tx patients in this step (see Supplementary File S1 for details).

**Table 4:** Survey results on the relevance of psychosocial problems before solid organ transplantation.

	Patients (*N* = 22)			Health care providers (*N* = 48)		
	Mean (SD)	“Very relevant” ratings	Missing values	Mean (SD)	“Very relevant” ratings	Missing values
Worries and anxieties						
General anxiety	2.27 (0.70)	—	—	3.37 (0.68)	22	2
Concern about the future	2.73 (0.99)	5	—	3.60 (0.50)	28	1
States of panic	1.64 (0.79)	—	—	2.81 (0.92)	13	1
Fears of mortality	1.95 (0.72)	—	—	3.35 (0.74)	23	2
Concern about the donor organ arriving in time	2.82 (0.96)	6	—	3.62 (0.61)	32	1
Fear of transplant surgery	2.09 (1.02)	2	—	3.19 (0.77)	19	1
Fear of post-transplant medical complications	2.59 (0.96)	4	—	3.11 (0.79)	16	1
Fear of infections	2.48 (0.87)	2	1	2.83 (0.92)	13	1
Psychological problems						
Lack of motivation	2.00 (0.93)	2	—	2.96 (0.82)	12	2
Feelings of futility	1.62 (0.74)	1	1	2.53 (0.87)	6	3
Listlessness	1.71 (0.64)	—	1	2.67 (0.85)	8	2
Glumness	2.00 (0.82)	—	—	3.26 (0.65)	16	2
Irritability	1.86 (0.83)	1	—	2.63 (0.77)	5	2
Self-perception						
Problems with self-esteem	1.67 (0.80)	—	1	2.51 (0.84)	5	3
Adjustment of life goals	2.12 (0.99)	2	5	3.19 (0.70)	15	5
Enduring sick role	2.05 (0.76)	—	2	3.18 (0.72)	16	3
Limits in self-determination	2.41 (0.96)	3	—	3.17 (0.83)	19	2
Waiting for the donor organ						
Strain due to the uncertain waiting period	2.50 (1.14)	5	—	3.74 (0.49)	36	1
Decisional conflict about the transplantation	1.32 (0.72)	—	—	2.72 (0.83)	10	1
Thoughts and feelings about the donor	1.59 (0.73)	—	—	2.73 (0.94)	12	3
Social problems						
Financial difficulties	1.59 (0.73)	—	—	2.80 (0.86)	11	2
Occupational difficulties	1.64 (0.79)	—	—	3.00 (0.75)	13	1
Loss of social life	1.77 (0.92)	1	—	3.19 (0.85)	21	1
Social support deficits	1.55 (0.80)	1	—	3.04 (0.86)	18	2
Strain on family and friends	2.50 (1.01)	4	—	3.30 (0.69)	20	1
Living with the illness						
Worsening of the general health condition	2.77 (0.75)	2	—	3.41 (0.58)	21	2
Severe physical discomforts	2.20 (0.83)	—	—	3.30 (0.67)	18	4
Problems with concentration	2.00 (0.76)	—	—	3.00 (0.79)	13	2
States of confusion	1.50 (0.74)	—	—	2.64 (0.94)	10	1
Exhaustion	2.55 (0.91)	3	—	3.32 (0.59)	18	1
Weakness	2.64 (0.85)	3	—	3.28 (0.65)	18	1
Sexual problems	1.77 (0.92)	2	—	2.76 (0.85)	11	2
Burden of medical treatment	2.14 (0.89)	1	—	3.21 (0.66)	16	1
Impaired coping in everyday life	2.64 (0.85)	3	—	3.28 (0.62)	17	2
Loss of autonomy	2.27 (1.03)	3	—	3.37 (0.65)	21	2
Adjustment of life style habits	2.19 (0.87)	1	1	3.17 (0.64)	14	2

**Table 5:** Survey results on the relevance of psychosocial problems after solid organ transplantation.

	Patients (*N* = 95)			Health care providers (*N* = 48)		
	Mean (SD)	“Very relevant” ratings	Missing values	Mean (SD)	“Very relevant” ratings	Missing values
Worries and anxieties						
General anxiety	1.89 (0.91)	6	6	3.14 (0.80)	15	5
Concern about the future	2.26 (1.06)	14	5	3.27 (0.67)	17	4
States of panic	1.54 (0.84)	4	2	2.50 (0.98)	9	4
Fear of organ rejection	2.39 (0.98)	14	1	3.66 (0.57)	31	4
Fear of re-transplantation	1.97 (1.00)	8	3	2.76 (0.91)	10	6
Fear of infections	2.72 (1.06)	26	1	3.41 (0.73)	24	4
Fear of side-effects	2.35 (0.94)	9	3	3.23 (0.75)	18	5
Self-perception						
Problems with self-esteem	1.73 (0.89)	6	9	2.64 (0.81)	8	4
Experience of being a different person	1.52 (0.82)	3	4	2.56 (0.96)	10	5
Relation to one’s body	2.03 (1.10)	11	19	2.88 (0.84)	11	7
Enduring sick role	2.26 (1.07)	15	14	2.79 (0.74)	7	5
Limits in self-determination	1.81 (0.95)	7	9	2.75 (0.87)	9	4
Psychological problems						
Lack of motivation	2.03 (0.94)	7	1	3.00 (0.78)	11	4
Feelings of futility	1.43 (0.79)	4	2	2.44 (0.93)	7	5
Listlessness	1.87 (0.92)	6	1	2.70 (0.88)	8	4
Glumness	1.96 (0.97)	11	2	2.91 (0.84)	11	5
Irritability	2.15 (1.00)	10	2	2.68 (0.74)	7	4
Nightmares	1.66 (0.92)	8	—	2.66 (0.97)	12	4
Flashbacks	1.74 (0.88)	4	9	2.95 (0.82)	13	5
Compulsions	1.49 (0.78)	3	10	2.23 (0.90)	5	5
Permanent feelings of tension	1.71 (0.84)	3	1	2.53 (0.80)	6	5
Focus on body symptoms	2.39 (1.01)	13	6	3.09 (0.77)	15	4
Social problems						
Conflicts with family and friends	1.89 (0.95)	6	—	2.95 (0.76)	10	6
Strains on family and friends	2.24 (1.09)	13	7	3.19 (0.76)	16	5
Social support deficits	1.60 (0.87)	6	1	2.79 (0.91)	13	5
Loss of social life	1.64 (0.87)	5	3	2.84 (0.87)	11	5
Financial difficulties	1.85 (0.95)	6	3	2.86 (0.89)	12	5
Occupational difficulties	1.78 (1.00)	5	2	3.00 (0.79)	13	5
Physical problems						
Drug side-effects	2.60 (0.86)	13	1	3.47 (0.63)	23	5
Medical complications	2.41 (0.91)	10	4	3.63 (0.54)	28	5
Infections	2.56 (1.06)	21	2	3.49 (0.70)	26	5
Pain	2.17 (0.94)	9	—	3.05 (0.73)	18	5
Exhaustion	2.48 (0.95)	15	1	3.24 (0.66)	15	6
Sleep disorders	2.36 (1.04)	18	—	3.05 (0.73)	12	6
Living with the transplant						
Frustration about the results of the surgery	1.42 (0.79)	3	3	2.95 (0.75)	10	8
Feelings of responsibility for the new organ	3.38 (0.96)	54	8	3.31 (0.72)	19	6
Mourning over the lost organ	1.56 (0.98)	8	4	1.93 (0.93)	4	7
Feelings of guilt	1.47 (0.72)	1	4	2.31 (0.89)	5	9
Ability to talk about the transplantation	2.22 (1.07)	10	10	3.21 (0.87)	19	6
Adjustment to the new situation	2.19 (1.09)	13	6	3.40 (0.66)	21	5
Difficulties with the treatment plan						
Daily medication schedule	2.10 (1.20)	21	1	3.40 (0.76)	24	5
Adjustment of life style habits	2.10 (0.99)	10	2	3.37 (0.73)	22	5
Regular medical surveillance	2.39 (1.20)	23	—	3.23 (0.87)	21	5
Conflicts with health care providers	1.39 (0.84)	6	—	2.79 (0.90)	12	6

### Final adaptation of the psychosocial problem lists based on comprehensibility ratings and qualitative analysis

In the following step, ratings on the clarity of psychosocial problems (see Tables 6, 7) and results from the qualitative analysis of the interviews and open response fields of the survey questionnaires were used to adapt the problem lists. Interviews with pre-Tx patients (*N* = 7) lasted for a mean of 3 min and 17 s (SD = 1 min 50 s, range: 1 min 19 s–6 min 48 s). Interviews with post-Tx patients (*N* = 51) lasted for a mean of 3 min and 33 s (SD = 2 min 21 s, range: 58 s–11 min 41 s). Interviews with transplant nurses lasted for a mean of 16 min and 17 s (SD = 18 min 52 s, range: 3 min 7 s–37 min 54 s). Categories used for the qualitative interview analysis were understanding of the problem, alternative wording of the problem, and missing problems. An overview of the adaptations and additions made to the problem lists is shown in Supplementary File S2. This resulted in lists of 21 psychosocial problems for patients before and 22 psychosocial problems for patients after solid organ transplantation. Psychosocial problems of both lists fit in the categories of *problems in everyday life*, *social problems*, *worries and anxieties*, and *physical and psychological problems*. The distress thermometer including the final problem lists are depicted in Supplementary File S3.

**Table 6:** Survey results on the clarity of psychosocial problems before solid organ transplantation.

	Patients (*N* = 22)		Health care providers (*N* = 48)	
	“Unclear” ratings	Missing values	“Unclear” ratings	Missing values
Worries and anxieties				
General anxiety	—	—	1	1
Concern about the future	—	—	—	1
States of panic	—	—	—	1
Fears of mortality	—	—	—	2
Concern about the donor organ arriving in time	—	—	—	1
Fear of transplant surgery	—	—	—	1
Fear of post-transplant medical complications	—	—	—	1
Fear of infections	—	1	—	1
Psychological problems				
Lack of motivation	—	—	1	1
Feelings of futility	1	—	2	1
Listlessness	1	—	1	1
Glumness	—	—	1	1
Irritability	—	—	1	1
Self-perception				
Problems with self-esteem	1	—	2	1
Adjustment of life goals	5	—	3	2
Enduring sick role	2	—	2	1
Limits in self-determination	—	—	1	1
Waiting for the donor organ				
Strain due to the uncertain waiting period	—	—	—	1
Decisional conflict about the transplantation	—	—	—	1
Thoughts and feelings about the donor	—	—	—	2
Social problems				
Financial difficulties	—	—	1	1
Occupational difficulties	—	—	—	1
Loss of social life	—	—	—	1
Social support deficits	—	—	—	1
Strains on family and friends	—	—	—	1
Living with the illness				
Worsening of the general health condition	—	—	1	1
Severe physical discomforts	2	—	2	1
Problems with concentration	—	—	—	2
States of confusion	—	—	—	1
Exhaustion	—	—	—	1
Weakness	—	—	—	1
Sexual problems	—	—	1	1
Burden of medical treatment	—	—	—	1
Impaired coping in everyday life	—	—	—	1
Loss of autonomy	—	—	—	2
Adjustment of life style habits	1	—	1	1

**Table 7:** Survey results on the clarity of psychosocial problems after solid organ transplantation.

	Patients (*N* = 95)		Health care providers (*N* = 48)	
	“Unclear” ratings	Missing values	“Unclear” ratings	Missing values
Worries and anxieties				
General anxiety	6	1	1	4
Concern about the future	2	3	—	4
States of panic	—	2	—	4
Fear of organ rejection	1	1	—	4
Fear of re-transplantation	6	1	1	5
Fear of infections	1	1	—	5
Fear of side-effects	2	1	—	4
Self-perception				
Problems with self-esteem	9	1	—	4
Experience of being a different person	2	2	—	4
Relation to one’s body	18	1	2	4
Enduring sick role	14	1	—	4
Limits in self-determination	9	1	—	4
Psychological problems				
Lack of motivation	1	—	—	4
Feelings of futility	5	—	1	4
Listlessness	—	1	—	4
Glumness	1	1	—	4
Irritability	1	1	—	4
Nightmares	—	—	—	4
Flashbacks	10	—	1	4
Compulsions	11	—	1	4
Permanent feelings of tension	1	—	1	4
Focus on body symptoms	6	—	—	4
Social problems				
Conflicts with family and friends	1	—	1	5
Strains on family and friends	8	1	1	5
Social support deficits	1	—	—	5
Loss of social life	2	1	—	5
Financial difficulties	2	1	—	5
Occupational difficulties	1	—	—	5
Physical problems				
Drug side-effects	1	—	—	5
Medical complications	4	—	—	5
Infections	1	1	—	5
Pain	—	—	—	5
Exhaustion	—	1	—	5
Sleep disorders	—	—	—	5
Living with the transplant				
Frustration about the results of the surgery	4	—	3	5
Feelings of responsibility for the new organ	7	1	—	5
Mourning over the lost organ	4	1	2	5
Feelings of guilt	1	3	3	6
Ability to talk about the transplantation	11	—	1	5
Adjustment to the new situation	5	1	—	5
Difficulties with the treatment plan				
Daily medication schedule	1	—	—	5
Adjustment of life style habits	2	—	—	5
Regular medical surveillance	—	—	—	5
Conflicts with health care providers	—	—	—	6

### Psychometric assessment

In total, 111 patients participated. As only eight transplant candidates could be reached, these were excluded from the analysis and only transplant recipients were included in the study. Three patients were excluded because they did not give information on the organ that was transplanted. For a description of the sample see Table 2. The organs patients received were lung (*n* = 11), heart (*n* = 57), kidney (*n* = 14) and liver (*n* = 18). Table 8 displays how patients after transplantation filled out the distress screening including the distress thermometer, the problem list and the measures of mental health.

**Table 8:** Results on the distress screening of transplant recipients.

Distress level^a^ (*n* = 84)	
Mean (SD)	4.5 (2.9)
Distress ≥4 [*n* (%)]	51 (60.7)
No. of problems (*n* = 100)	
Mean (SD) range	6.0 (3.7) 0–16
Frequency of problems	
Problems in everyday life	*n* (%)
Regular medical surveillance (*n* = 96)	22 (22.9)
Daily medication schedule (*n* = 99)	19 (19.2)
Adjustment of lifestyle habits (*n* = 94)	18 (19.1)
Burden of responsibility for the new organ (*n* = 97)	7 (7.2)
Fact of never being fully well again (*n* = 100)	23 (23)
Social problems	
Feeling of being a burden to others (*n* = 97)	22 (22.7)
Worries about family and friends (*n* = 97)	42 (43.3)
Difficulties in talking about the transplantation (*n* = 97)	5 (5.2)
Occupational difficulties (*n* = 87)	13 (14.9)
Lack of support in the health care system (*n* = 97)	19 (19.6)
Worries and anxieties	
About the future (*n* = 96)	31 (32.3)
About drug side-effects (*n* = 96)	33 (34.4)
About infections (*n* = 98)	48 (49.0)
About transplant rejection and the need for a repeat transplantation (*n* = 95)	40 (42.1)
Physical and psychological problems	
Exhaustion, mental or physical (*n* = 92)	43 (46.7)
Sleep disorders (*n* = 95)	40 (42.1)
Sexual problems (*n* = 87)	18 (20.7)
Increased focus on body symptoms (*n* = 92)	39 (42.4)
Pain (*n* = 95)	36 (37.9)
Infections (*n* = 91)	24 (26.4)
Drug side-effects (*n* = 95)	35 (36.8)
Medical complications and transplant-induced illnesses (*n* = 93)	29 (31.2)
PHQ-4 (*n* = 94)	
Normal (0–2)	63 (67.0)
Mild (3–5)	19 (20.2)
Moderate (6–8)	7 (7.5)
Severe (9–12)	5 (5.3)
PHQ-2 (*n* = 96)	
<2	83 (86.5)
≥3	13 (13.5)
GAD-2 (*n* = 97)	
<2	85 (87.6)
≥3	12 (12.4)
Asked to see a therapist (*n* = 97)	
Yes	18 (18.6)

a Scale 0 (no distress) to 10 (extreme distress).

### Concurrent validity

The correlations of the distress level of patients after the transplant with different mental health measures are displayed in Table 9. Also the concurrent validity of the problem list assessed by correlating the number of problems endorsed after transplantation with the mental health measures is demonstrated in Table 9.

**Table 9:** Correlations between distress and the number of problems and mental health measures of transplant recipients.

		Distress	PHQ-4	PHQ-2	GAD-2	General health	Mental health	Need to talk
No. of problems	*r*	0.41^**^	0.63^**^	0.53^**^	0.60^**^	−0.42^**^	−0.50^**^	0.39^**^
	*n*	84	94	96	97	98	99	97
Distress	*r*	1	0.54^**^	0.56^**^	0.45^**^	−0.36^**^	−0.51^**^	0.34^*^
	*n*	84	82	82	84	84	84	82

^*^p < 0.01 and ^**^p < 0.001.

## Discussion

The goal of this study was the content-valid development of two specific problem lists for transplant candidates and recipients in order to obtain a short and easily applicable screening tool which assesses their level of psychosocial distress. To our knowledge, this is the first attempt to develop a specific problem lists to cover psychosocial distress of these patients. Out of 36 (44) items derived from the literature, 21 (22) items have been selected in a systematic process with qualitative and quantitative steps including the patients’ as well as HCPs’ perspectives. Both lists include problems that fit in the categories of problems in everyday life, social problems, worries and anxieties, and physical and psychological problems.

Recent literature supports a substantial impact of immunosuppressive treatment on physical symptoms and many aspects of daily living (Demir and Bulbuloglu, 2021; Bulbuloglu et al., 2022). In our screening, problems with medication are assessed from the patients’ perspective. Although medication adherence to immunosuppressive therapy is very important especially for transplant recipients, it was not represented in the screening tool. In our view, medication adherence should be considered as outcome or mediator of long term morbidity and mortality. There are existing measures of medication adherence. Furthermore, it has been recommended to assess medication adherence using multiple sources (Dobbels et al., 2010). Psychosocial distress, which is assessed in our tool, can be seen as one possible predictor of problems with medication adherence but our screening does not aim to assess adherence itself.

The problem list for transplant recipients was piloted as part of a distress screening. The results show that each problem was endorsed by some patients and that the list has a good concurrent validity.

The problem lists in combination with the distress thermometer can be used as screening tools in routine care and facilitate early detection of psychosocial distress and mental disorders.

Across all items on the candidates and recipient lists HCPs rated the problems as more relevant than the patients and there were more “very relevant” ratings of HCPs on almost every item, especially for transplant candidates. A possible explanation could be that patients have adapted their expectations to their illness and changed their internal standards for evaluating their health. A similar response shift was reported earlier in transplant patients and patients with other illnesses (Adang et al., 1998; Ubel et al., 2003). Besides, patients’ assessments depend on the timing when they fill out the problem list. Anxiety and worries are likely to increase the longer a patient waits for an organ. After the transplantation anxiety and other difficulties may decrease over time while depressive symptoms have been reported to increase (Dobbels et al., 2006; Dew et al., 2015; Schulz and Kroencke, 2015). Prevalence of psychological problems are highest in the first 2 years after transplantation (Annema et al., 2015). Patients’ evaluations are affected by their current health whereas HCPs base their assessment on their overall experience with transplant patients (Ubel et al., 2003).

The only item that was rated as more relevant by patients as compared to HCPs was *feelings of responsibility for the new organ*. The item also received more than twice as many “very relevant” ratings from patients. Patients pre-and post-transplantation rated items from the categories “worries and anxieties” and “physical and psychological problems” as especially relevant. Similarly, in oncology patients worry was the item strongest associated with distress. Those at risk for high distress were more than five times more likely to report worrying (VanHoose et al., 2015). Other frequently endorsed items were from the emotional and physical domain (VanHoose et al., 2015). Although the items on the list before and after transplantation are similar to a certain extent, we recommend maintaining two separate problem lists. The stressors and challenges before and after a transplantation differ substantially (Schulz and Kroencke, 2015). This cannot be represented sufficiently in a single list.

The results of the piloting of the distress screening demonstrate in line with previous findings that patients after transplantation show high levels of distress. More than half of the patients reported a distress score of 4 or higher which had been identified as a cut-off score for clinically significant distress in oncology patients (Jacobsen et al., 2005; Donovan et al., 2014). However, Donovan and Grassi (25) note that different patient groups might need different cut-off scores and the ideal cut-off score for transplant patients has yet to be determined.

The patients especially endorsed items concerning worries and anxiety on the problem list. Similarly, Ivarsson et al. (2011) found that patients are especially worried about the future. Despite the high levels of distress less than 20% of patients wanted to talk to a psychologist. Previous research also found that some highly distressed people indicate no need for services (Carlson et al., 2004; van Scheppingen et al., 2011; Faller et al., 2016). Carlson found that the main reason for not wanting any service was a perception of not needing any help (Carlson et al., 2004). A need for help was associated with younger age and female sex as well as not being married and living alone (Faller et al., 2016). Many patients prefer talking to a family member (Faller et al., 2016).

The correlation analyses demonstrate that the number of problems on the problem list correlate with a range of mental health measures such as the well-validated PHQ-4, PHQ-2, and GAD-2 (Lowe et al., 2010). The number of problems also correlate with the level of distress during the past week indicated on the NCCN distress thermometer (Donovan et al., 2014). This measure of distress has been validated as being suitable for determining distress in patients (Mitchell, 2010). Thus, the problem list especially in combination with the distress thermometer appears to have good concurrent validity and is appropriate for identifying patients with high levels of distress or a predisposition for mental health problems after the transplantation. However, these findings need to be corroborated in further studies.

As the problems contained in the list are not considered to reflect on a latent variable but instead are a formative measure which should be seen as the source of distress other psychometric measure such as internal consistency are not suitable in this context. Therefore, the answers of the participants cannot be summed up to a single score. Each problem endorsed by the patients should be considered as contributing to the individuals’ distress and can be the used as the starting point for an intervention.

This study is subject to some limitations. In our developmental process and definition of content validity, we referred to the COSMIN standards and definitions (Terwee et al., 2018). Since there are also other existing recommendations regarding content valid scale development (e.g., Boateng et al., 2018; Patrick et al., 2011), its use may have resulted in slightly different procedures and results.

This is a single center study, furthermore we did not include expertise from other countries. The literature search has been conducted as first step of the project in 2019 and therefore does not include more recent literature regarding the situation of transplant patients (e.g., Bulbuloglu and Demir, 2021; Harmancı et al., 2023; Saritaş et al., 2024). Our literature search was limited to PubMed, we therefore may have missed publications that are recorded in other databases only. Also, the list was only assessed by German speaking patients. It is possible that patients whose first language is not German will encounter different problems such as lack of support due to language barriers or limited understanding from friends because of insufficient information in other languages. Also, the sample was highly educated which reduces the diversity of the group further. For all these reasons it is likely, that the list might need adaptation when implemented in a different setting, patient group or country.

Furthermore, we only reached a small sample of HCPs and nurses that participated in the study. HCPs did not elaborate on why they would not participate. However, it seems likely that it is due to time constraints and limited resources. Only a small sample of patients assessed the problem lists for transplant candidates in the first part of the study. In the psychometric analyses, data from transplant candidates could not be included at all. In sum, transplant candidates could not be reached sufficiently during the course of this study. Therefore, we cannot make any conclusions about the concurrent validity of the problem list for transplant candidates.

## Conclusions and outlook

Despite these limitations, this study provides important tools for the identification of psychosocial distress of transplant candidates and recipients. The developed screening tools can be an effective instrument for improving the communication between HCPs and patients about their mental health. Face validity and initial content validity were established by relevance assessments of patients and HCPs. Concurrent validity could be obtained for the problem list for transplant recipients. We recommend using the problem list in combination with components comparable to those employed in this study, i.e., the distress thermometer and the PHQ-4. Future studies should include a psychometric evaluation using larger and more diverse samples, determine the sensitivity and specificity of the screening, identify an appropriate cut-off score for this population and specify the necessary interventions that should follow different results of the screening. Due to the lack of valid data gathered in this study, special attention should be paid to the problem list for transplant candidates.

## Data Availability

The raw data supporting the conclusions of this article will be made available by the authors, without undue reservation.
