# Supplementary material for: The development and psychometric evaluation of specific problem lists reflecting psychosocial distress of patients before and after solid organ transplantation
Source: Front Psychol. 2025 May 13;16:1481641. doi: 10.3389/fpsyg.2025.1481641 (PMC12106308; doi:10.3389/fpsyg.2025.1481641)
Supplement: Supplementary file 1 [file Supplementary_file_1.docx]

**Final list of 22 psychosocial problems for transplant recipients**

**20 problems in 4 categories:** *Worries and anxieties* (concern about the future, fear of organ rejection, fear of infections, fear of side-effects), *Physical problems* (drug side-effects, medical complications, infections, pain, exhaustion, sleep disorders, focus on body symptoms), *Social problems (*strains on family and friends, occupational difficulties, ability to talk about the transplantation), *Problems in everyday life* (adjustment to the new situation, adjustment of life style habits, daily medication schedule, regular medical surveillance, feelings of responsibility for the new organ, fact of never being fully well again)

**20 problems in 4 categories:** *Worries and anxieties* (concern about the future, concern about the donor organ arriving in time, fear of post-transplant medical complications, fears of mortality, fear of transplant surgery), *Physical problems (*worsening of the general health condition, exhaustion, weakness, sexual problems, severe physical discomforts), *Social problems (*loss of social life, social support deficits, strain on family and friends), *Problems in everyday life (*burden of the uncertain waiting period, burden of medical treatment, fact of never being fully well again, impaired coping in everyday life, loss of autonomy, adjustment of life style habits, limits in self-determination)

***Quantitative analysis of survey data***

(Two of the following criteria should be met: 1. a minimum of 15 HCPs rated the problem as “very relevant”, 2. the mean of HCP relevance ratings reached 3 or more, 3. a minimum of 10% of pre- or post-Tx patients, respectively, rated the problem as “very relevant”, 4. the mean of patient relevance ratings reached 2.5 or more, 5. the mean of young (< 40 years old) patient relevance ratings reached 2.5 or more)

***Qualitative analysis of interview transcripts and open response fields***

(to identify missing problems and adapt problems that were rated as unclear)

**Final list of 21 psychosocial problems for transplant candidates**

**36 problems in 6 categories**: *Worries and anxieties* (general anxiety, concern about the future, states of panic, fears of mortality, concern about the donor organ arriving in time, fear of transplant surgery, fear of post-transplant medical complications, fear of infections), *Psychological problems* (lack of motivation, feelings of futility, listlessness, glumness, irritability), *Self-perception* (problems with self-esteem, adjustment of life goals, enduring sick role, limits in self-determination), *Waiting for the donor organ* (burden of the uncertain waiting period, decisional conflict about the transplantation, thoughts and feelings about the donor), *Social problems* (financial difficulties, occupational difficulties, loss of social life, social support deficits, strain on family and friends)*, Living with the illness* (worsening of the general health condition, severe physical discomforts, problems with concentration, states of confusion, exhaustion, weakness, sexual problems, burden of medical treatment, impaired coping in everyday life, loss of autonomy, adjustment of life style habits)

**44 problems in 7 categories**: *Worries and anxieties* (general anxiety, concern about the future, states of panic, fear of organ rejection, fear of re-transplantation, fear of infections, fear of side-effects), *Self-perception* (problems with self-esteem, experience of being a different person, relation to one’s body, enduring sick role, limits in self-determination), *Psychological problems* (lack of motivation, feeling of futility, listlessness, glumness, irritability, nightmares, flashbacks, compulsions, permanent feelings of tension, focus on body symptoms), *Social problems* (conflicts with family and friends, strains on family and friends, social support deficits, loss of social life, financial difficulties, occupational difficulties), *Physical problems* (drug side-effects, medical complications, infections, pain, exhaustion, sleep disorders), *Living with the transplant* (frustration about the result of the surgery, feelings of responsibility for the new organ, mourning over the lost organ, feelings of guilt, ability to talk about the transplantation, adjustment to the new situation), *Difficulties with the treatment plan* (daily medication schedule, adjustment of life style habits, regular medical surveillance, conflicts with health care providers)

***Transplantation recipients***

***Transplantation candidates***
