# Supplementary material for: The development and psychometric evaluation of specific problem lists reflecting psychosocial distress of patients before and after solid organ transplantation
Source: Front Psychol. 2025 May 13;16:1481641. doi: 10.3389/fpsyg.2025.1481641 (PMC12106308; doi:10.3389/fpsyg.2025.1481641)
Supplement: Supplementary file 2 [file Supplementary_file_2.docx]

**Supplementary File S2: Adaptation of the psychosocial problem lists based on clarity ratings and qualitative analysis**

Table S1. Adaptation of the psychosocial problem list before solid organ transplantation

|  | **Adaptations or additions** | **Reasons for adaptations or additions** |
| --- | --- | --- |
| **Problems in everyday life** |  |  |
| Burden of the uncertain waiting period | - | - |
| Burden of medical treatment | - | - |
| Fact of never being fully well again | Adaptation of the original “enduring sick role” | Two transplant candidates and four transplant recipients rated the original as unclear interview results showed that patients considered the fact of being chronically ill important |
| Impaired coping in everyday life | - | - |
| Adjustment of life goals to the new situation | Adaptation of the original “adjustment of life goals” | Five transplant candidates patients and three HCPs rated the original as unclear |
| **Social problems** |  |  |
| Feeling of being a burden to others | Adaptation of the original “strains on family and friends” | Eight transplant recipients rated the original as unclear and interview results indicated that two different aspects in relation to family and friends were important |
| Worries about family and friends | Adaptation of the original “strains on family and friends” |  |
| Loss of social life | - | - |
| Social support deficits | - | - |
| Lack of support in the health care system | Addition | transplant candidates and recipients mentioned missing problems that fit this problem |
| **Worries and anxieties** |  |  |
| about the future | Adaptation of the original “concern about the future” | One patient suggested to mitigate the term “fear” |
| about the donor organ arriving in time | Adaptation of the original “concern about the donor organ arriving in time” |  |
| about mortality | Adaptation of the original “fears of mortality” |  |
| about the transplant surgery | Adaptation of the original “fear of transplant surgery” |  |
| about post-transplant medical complications | Adaptation of the original “fear of post-transplant medical complications” |  |
| **Physical and psychological problems** |  |  |
| Worsening of the general health condition | - | - |
| Sleep disorders | Addition | One HCP mentioned “sleep disorders” as a missing problem for transplant candidates; Transplant recipients and HCPs considered “sleep disorders” relevant |
| Exhaustion, mental or physical | Adaptation of the originals “exhaustion” and “weakness” | One HCP considered “exhaustion” and “weakness” similar; one patient indicated that “exhaustion” can be mental or physical |
| Feelings of loss of control | Adaptation of the original “loss of autonomy” | One HCP mentioned “loss of control” as a missing problem |
| Sexual problems | - | “Sexual problems” did not meet required relevance criteria, but 10% transplant candidates considered them very relevant and one HCP indicated its high relevance for transplant patients in the interview |
| Severe physical discomforts and limitations | Adaptation of the original “severe physical discomforts” | Two transplant candidates and two HCPs rated the original as unclear; One patient considered “physical limitations” important |

Table S2. Adaptation of the psychosocial problem list after solid organ transplantation

|  | **Adaptations or additions** | **Reasons for adaptations or additions** |
| --- | --- | --- |
| **problems in everyday life** |  |  |
| regular medical surveillance | - | - |
| daily medication schedule | - | - |
| adjustment of life style habits | - | - |
| burden of responsibility for the new organ | Adaptation of the original “feelings of responsibility for the new organ” | Seven transplant recipients rated the original as unclear and interview results revealed that the negative impact of the feelings of responsibility was unclear |
| fact of never being fully well again | Adaptation of the original “enduring sick role” | Fourteen transplant recipients rated the original as unclear and interview results showed that patients considered the fact of being chronically ill important |
| **social problems** |  |  |
| feeling of being a burden to others | Adaptation of the original “strains on family and friends” | Eight transplant recipients rated the original as unclear and interview results indicated that two different aspects in relation to family and friends were important |
| worries about family and friends | Adaptation of the original “strains on family and friends” |  |
| difficulties in talking about the transplantation | Adaptation of the original “ability to talk about the transplantation” | Eleven transplant recipients and one HCP rated the original as unclear and one patient suggested this alternative wording |
| occupational difficulties | - | - |
| lack of support in the health care system | Addition | transplant candidates and recipients mentioned missing problems that fit this problem |
| **worries and anxieties** |  |  |
| about the future | Adaptation of the original “concern about the future” | One patient suggested to mitigate the term “fear” |
| about drug side-effects | Adaptation of the original “fear of side-effects” |  |
| about infections | Adaptation of the original “fear of infections” |  |
| about transplant rejection and the need for a repeat transplantation | Adaptation and integration of the originals “fear of organ rejection” and “fear of re-transplantation” | Six transplant recipients rated the original “re-transplantation” as unclear and two patients suggested to use “repeat transplantation” instead. |
| **physical and psychological problems** |  |  |
| exhaustion, mental or physical | Adaptation of the original “exhaustion” | One patient indicated that “exhaustion” can be mental or physical |
| sleep disorders | - | - |
| sexual problems | Addition | “Sexual problems” were not part of the original list, but one HCP indicated its high relevance for transplant patients in the interview |
| increased focus on body symptoms | Adaptation of the original “focus on body symptoms” | Six transplant recipients rated the original as unclear. |
| pain | - | - |
| infections | - | - |
| drug side-effects | - | - |
| medical complications and transplant-induced illnesses | Adaptation of the original “medical complications” | Four transplant recipients rated the original as unclear und two patients suggested the alternative wording of transplant-induced illnesses |
