# Supplementary material for: The development and psychometric evaluation of specific problem lists reflecting psychosocial distress of patients before and after solid organ transplantation
Source: Front Psychol. 2025 May 13;16:1481641. doi: 10.3389/fpsyg.2025.1481641 (PMC12106308; doi:10.3389/fpsyg.2025.1481641)
Supplement: Supplementary file 3 [file Supplementary_file_3.docx]

**Supplementary File S3: Distress thermometer and psychosocial problem lists**

**Distress Thermometer and list of psychosocial problems for transplant candidates**

**Other problems:**

**1.** Please circle the number (0-10) that best describes how much distress you have been experiencing in the past week including today.

**Extreme distress**

**No distress**


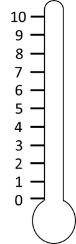


**2.** Please indicate if any of the following has been a problem for you in the past week including today. Be sure to

check YES or NO for each.

| **Yes** | **No** |  |
| --- | --- | --- |
|  |  | **problems in everyday life** |
| ⃝ | ⃝ | burden of the uncertain waiting period |
| ⃝ | ⃝ | burden of medical treatment |
| ⃝ | ⃝ | fact of never being fully well again |
| ⃝ | ⃝ | impaired coping in everyday life |
| ⃝ | ⃝ | adjustment of life goals to the new situation |
|  |  | **social problems** |
| ⃝ | ⃝ | feeling of being a burden to others |
| ⃝ | ⃝ | worries about family and friends |
| ⃝ | ⃝ | loss of social life |
| ⃝ | ⃝ | social support deficits |
| ⃝ | ⃝ | lack of support in the health care system |

| **Yes** | **No** |  |
| --- | --- | --- |
|  |  | **worries and anxieties** |
| ⃝ | ⃝ | about the future |
| ⃝ | ⃝ | about the donor organ arriving in time |
| ⃝ | ⃝ | about mortality |
| ⃝ | ⃝ | about the transplant surgery |
| ⃝ | ⃝ | about post-transplant medical complications |
|  |  | **physical and psychological problems** |
| ⃝ | ⃝ | worsening of the general health condition |
| ⃝ | ⃝ | sleep disorders |
| ⃝ | ⃝ | exhaustion, mental or physical |
| ⃝ | ⃝ | feelings of loss of control |
| ⃝ | ⃝ | sexual problems |
| ⃝ | ⃝ | severe physical discomforts and limitations |

**Distress Thermometer and list of psychosocial problems for transplant recipients**

**Other problems:**

**1.** Please circle the number (0-10) that best describes how much distress you have been experiencing in the past week including today.

**Extreme distress**

**No distress**


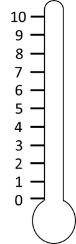


**2.** Please indicate if any of the following has been a problem for you in the past week including today. Be sure to

check YES or NO for each.

| **YES** | **NO** |  |
| --- | --- | --- |
|  |  | **problems in everyday life** |
| ⃝ | ⃝ | regular medical surveillance |
| ⃝ | ⃝ | daily medication schedule |
| ⃝ | ⃝ | adjustment of life style habits |
| ⃝ | ⃝ | burden of responsibility for the new organ |
| ⃝ | ⃝ | fact of never being fully well again |
|  |  | **social problems** |
| ⃝ | ⃝ | feeling of being a burden to others |
| ⃝ | ⃝ | worries about family and friends |
| ⃝ | ⃝ | difficulties in talking about the transplantation |
| ⃝ | ⃝ | occupational difficulties |
| ⃝ | ⃝ | lack of support in the health care system |

| **YES** | **NO** |  |
| --- | --- | --- |
|  |  | **worries and anxieties** |
| ⃝ | ⃝ | about the future |
| ⃝ | ⃝ | about drug side-effects |
| ⃝ | ⃝ | about infections |
| ⃝ | ⃝ | about transplant rejection and the need for a repeat transplantation |
|  |  | **physical and psychological problems** |
| ⃝ | ⃝ | exhaustion, mental or physical |
| ⃝ | ⃝ | sleep disorders |
| ⃝ | ⃝ | sexual problems |
| ⃝ | ⃝ | increased focus on body symptoms |
| ⃝ | ⃝ | pain |
| ⃝ | ⃝ | infections |
| ⃝ | ⃝ | drug side-effects |
| ⃝ | ⃝ | medical complications and transplant-induced illnesses |
